# PolyQ Expansion Controls Biomolecular Condensation and Aggregation of the N-Terminal Fragments of Ataxin-2

**DOI:** 10.3390/ijms262311538

**Published:** 2025-11-28

**Authors:** Yin-Hu Liu, Heng-Tong Duan, Lei-Lei Jiang, Hong-Yu Hu

**Affiliations:** 1 Key Laboratory of RNA Innovation, Science and Engineering, Center for Excellence in Molecular Cell Science, Shanghai Institute of Biochemistry and Cell Biology, Chinese Academy of Sciences, Shanghai 200031, China; liuyinhu2021@sibcb.ac.cn (Y.-H.L.); duanhengtong2021@sibcb.ac.cn (H.-T.D.); jiangleilei@sibcb.ac.cn (L.-L.J.); 2 University of Chinese Academy of Sciences, Beijing 100049, China

**Keywords:** ataxin-2, polyQ expansion, biomolecular condensation, aggregation, molecular dynamics

## Abstract

Ataxin-2 (Atx2) is a general RNA-binding protein involved in processes such as RNA processing and metabolism in cells. Atx2 is also a polyglutamine (polyQ) tract-containing protein; its abnormal expansion can lead to protein aggregation associated with neurodegenerative diseases. Previous studies have shown that the C-terminal intrinsically disordered regions (c-IDRs) of Atx2 participate in its condensation and aggregation processes. To elucidate the role of polyQ expansion in biomolecular condensation and aggregation, we studied the N-terminal fragments of Atx2 (namely, Atx2-N317 and Atx2-N81) that preserve a polyQ tract and compared their molecular behaviors in cells to those of the full-length Atx2. We found that the molecular mobility of the N-terminal fragments decreases with the increasing length of polyQ, indicating that polyQ expansion promotes a gradual phase transition to an irreversible and insoluble state. Moreover, the molecular state and mobility of Atx2-N317 are not distinct from those of Atx2-N81, regardless of the presence of other domains, demonstrating that the polyQ tract is a direct and sufficient element for protein condensation and aggregation, while the Like Sm (LSm) and LSm-associated (LSmAD) domains and their interactions with RNA are not necessary for these processes. This result is also validated through the in vitro investigation of Atx2-N81 with different polyQ expansions. This study reveals that polyQ expansion controls the biomolecular condensation and aggregation of the N-terminal fragments of Atx2 and is thus thought to modulate the dynamic behaviors of the full-length protein as well, which is implicated in the pathological accumulation of Atx2 in cells.

## 1. Introduction

Protein aggregation is one of the core pathological mechanisms of neurodegenerative diseases (NDDs). Among these, polyglutamine (polyQ) diseases are generally caused by the abnormal amplification of CAG trinucleotides in specific genes [1], characterized by the presence of excessively long polyQ sequences in the mutant proteins [2]. PolyQ-expanded proteins, such as huntingtin (Htt), form β-sheet folded structures through hydrophobic interactions and hydrogen bonding, triggering the nucleation and elongation of amyloid fibrils [3]. Protein phase separation or condensation is an important recent discovery in the field of cell biology and biophysics [4]. It refers to the spontaneous condensation of proteins or multi-component macromolecules under specific conditions to form a concentrated liquid or droplet phase, forming a physical boundary with the surrounding environment [5]. This process is widely involved in the formation of membrane-less organelles, such as stress granules (SGs), nucleoli, and processing bodies (P-bodies) [6], and plays a key role in gene regulation, signal transduction, and cellular stress response [7,8]. We have proposed that these biomolecular assemblies (condensates and aggregates) are able to sequester cellular essential factors and deplete their bioavailability [9], which is associated with cellular homeostasis and the pathogenic mechanism [10].

The molecular basis of protein condensation depends on the multivalent interactions of proteins [11], such as weak electrostatic [12] or hydrophobic interactions [13] in intrinsically disordered regions (IDRs) [14], and the synergistic binding of modular domains, such as SH3 [15]. Under some physiological situations, proteins undergo phase separation, forming dynamic droplets [6,16]. However, under pathological conditions [17], such as mutations, the protein condensates may further evolve into solid-phase or amyloid-like aggregates, leading to irreversible phase transitions [10]. For example, in amyotrophic lateral sclerosis (ALS) disease, the pathological aggregation of RNA-binding proteins (RBPs), such as TDP-43 [18] and FUS [19], is closely related to the abnormal phase transition to insoluble aggregates.

Ataxin-2 (Atx2) is such a polyQ tract-containing RBP [20] that may undergo biomolecular condensation and aggregation during its biological process and function [21]. Atx2 is widely expressed in eukaryotic cells and plays an important role in mRNA metabolism and translation regulation [22]. It is a component of various membrane-less organelles in cells, such as SGs [23] and P-bodies [24,25]. Its N-terminus contains a polyQ tract that may also trigger protein condensation and aggregation [26], and the Like Sm (LSm) and LSm-associated (LSmAD) domains that interact with RNA [27,28]. While its C-terminal portion is mainly comprised of low-complexity domains (LCDs), a PAM2 motif embedded in the LCD regions can bind PABPC1 [23,29], making it easy to participate in protein–protein/RNA interactions [30]. Under normal circumstances, Atx2 dynamically regulates RNA metabolism and cellular stress response through a phase separation mechanism [21,31]. However, when the CAG trinucleotide repeats in the *ATXN2* gene are abnormally amplified to an intermediate length (>23), it can lead to ALS pathology [32,33,34]; when the CAG is further abnormally amplified (>33), it may cause spinocerebellar ataxia type 2 (SCA2) [35,36]. The longer the polyQ expansion in Atx2, the earlier and more severe onset of SCA2 occurs [37,38,39].

In recent years, research on the *Drosophila* homolog has shown that the C-terminal IDRs (c-IDRs) are necessary for the localization of Atx2 in SGs [31]. However, there is still limited knowledge on the polyQ-tract impact on the phase separation (condensation) and phase transition in its N-terminal IDRs (n-IDRs), where abnormal polyQ expansion may presumably lead to the accumulation and precipitation of the protein. We propose that the N-terminal polyQ tract of Atx2 is equally crucial for its biomolecular condensation and aggregation. Under abnormal conditions of polyQ expansion, the irreversible phase transition of Atx2 from a soluble to an insoluble state plays an important role in the NDD pathology. So, in this study, we investigated the effects of polyQ expansion on the biomolecular aggregation of the N-terminal fragments of Atx2 and compared our results to those of studies on the full-length protein.

## 2. Results

### 2.1. PolyQ Expansion Reduces the Molecular Mobility of Atx2-N317 in Cells

There are some reports showing that Atx2 can lead to the occurrence of ALS when its polyQ tract expands abnormally at an intermediate length (29Q–33Q) [34], and more extensive polyQ expansion results in a liquid–solid phase transition in cells that may cause pathogenesis of SCA2 [35]. To investigate the impact of polyQ expansion on the molecular dynamics of Atx2, we designed a series of EGFP-tagged constructs of the N-terminal 317-residue fragments of Atx2, namely, Atx2-N317 [26], containing 9Q, 23Q, 33Q, and 41Q, respectively. Atx2-N317 is an N-terminal truncation of Atx2, mainly composed of the polyQ tract and the LSm-LSmAD domains (Figure S1) [28]. Our previous study revealed that polyQ-expanded Atx2-N317, as well as full-length Atx2, could form protein aggregates in cells and sequester DDX6 into these aggregates [26]. This implies that the N-terminal truncations can be used as a simple model for studying the impact of polyQ expansion on the condensation and aggregation behaviors of the Atx2 protein.

**Figure 1:**
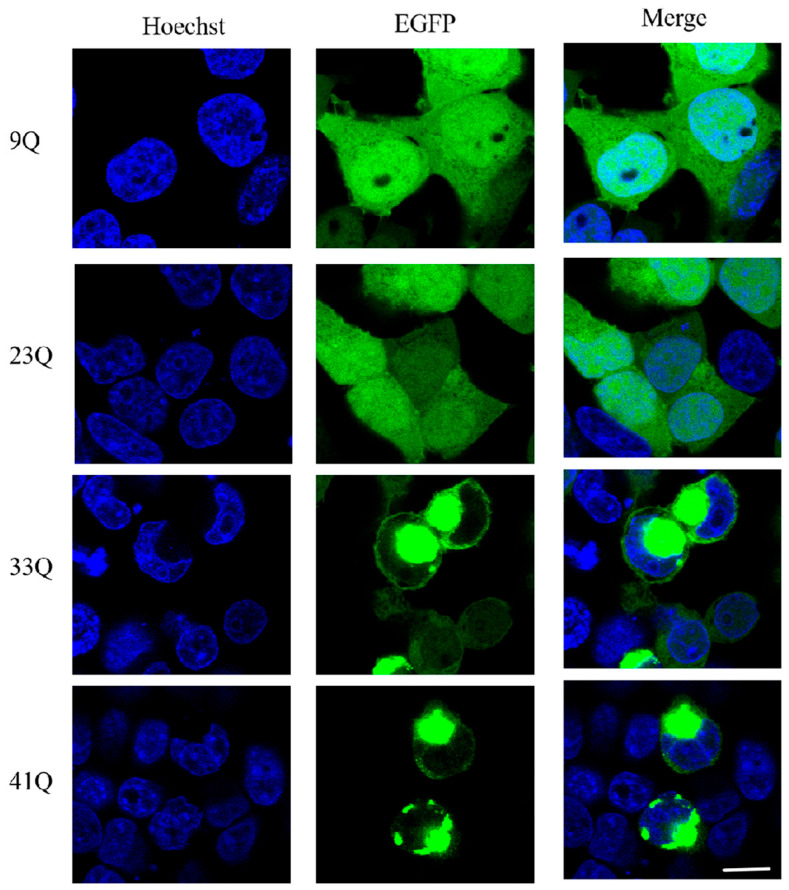
Immunofluorescence imaging showing the status and subcellular distribution of Atx2-N317 with different polyQ expansions. About 48 h after the transfection of the respective EGFP-Atx2-N317 plasmid in HEK 293T cells, the cells were fixed and photographed. Green, Atx2-N317 imaged with EGFP; blue, nuclei stained with Hoechst. Scale bar, 10 μm. 9Q, EGFP-Atx2_9Q_-N317; 23Q, EGFP-Atx2_23Q_-N317; 33Q, EGFP-Atx2_33Q_-N317; 41Q, EGFP-Atx2_41Q_-N317.

We first over-expressed the respective EGFP-tagged Atx2-N317 in HEK 293T cells and visualized their status by using confocal fluorescence microscopy (Figure 1). The experimental results showed that the Atx2-N317 proteins with short polyQ were distributed in both the nucleus and cytoplasm. The 9Q variant exhibited a completely diffusive distribution, possibly due to the solubilizing effect of the EGFP tag. The 23Q also showed diffusive distribution but with some small puncta in the cytoplasm. In contrast, with the expansion of the polyQ tract, the 33Q and 41Q proteins were only observed in the cytoplasm and mostly formed large aggregated puncta.

**Figure 2:**
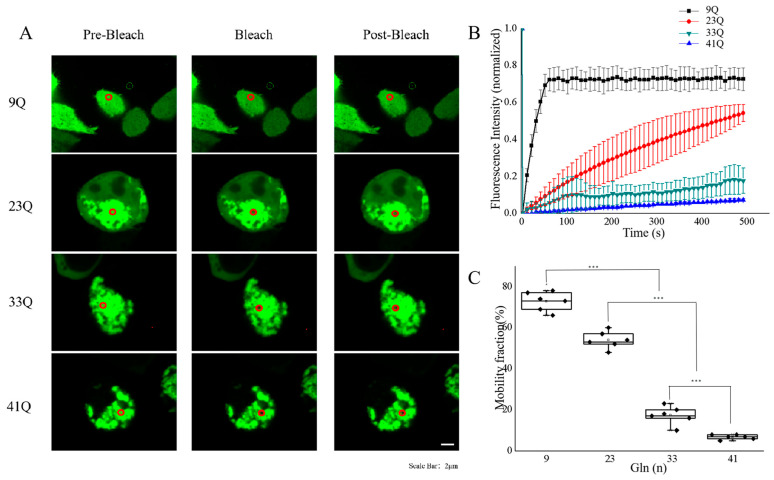
Molecular mobilities of Atx2-N317 with different polyQ expansions. (**A**) FRAP analysis for the molecular mobilities of Atx2-N317 with different polyQ lengths. HEK 293T cells were transfected with the respective EGFP-Atx2-N317 plasmids, and after a 48 h culture, the cells were subjected to FRAP experiments. The red circle represents the range of photobleaching. The changes in fluorescence intensity at the bleaching site were recorded after photobleaching, and the time was set as the starting point. Scale bar, 2 μm. 9Q, EGFP-Atx2_9Q_-N317; 23Q, EGFP-Atx2_23Q_-N317; 33Q, EGFP-Atx2_33Q_-N317; 41Q, EGFP-Atx2_41Q_-N317. (**B**) Fluorescence recovery profile after photobleaching. The curve shows the fluorescence intensity recovering at the bleaching site over time, normalized with the fluorescence intensity of the spot before bleaching. (**C**) Fluorescence recovery rate of photobleaching. Data are derived from three repeats and are represented as the Mean ± SEM. Statistical analysis was carried out with ANOVA. ***, *p* < 0.001.

To investigate the mobility of punctum-like particles formed by Atx2-N317 with different polyQ expansions in cells, we performed fluorescence recovery after a photobleaching (FRAP) experiment on the puncta formed by various Atx2-N317 species (Figure 2A). The Atx2_9Q_-N317 protein was diffusely distributed in cells, and the fluorescence bleaching at any spots could be quickly restored. The Atx2_23Q_-N317 protein was distributed in cells with two different morphologies: diffused dots and puncta. We selected the punctum-like spots formed by Atx2_23Q_-N317 in cells and bleached the region of interest (ROI), which could only reflect the mobility feature of the punctum-like granules but not the diffused fractions. After 300 s, the fluorescence intensity at the ROI could recover to about 60% (Figure 2B,C), while those of the 33Q and 41Q species were only 5–10%, with the 41Q showing almost no fluorescence recovery. These results suggest that the molecular mobility of the Atx2-N317 protein decreases with the expansion of the polyQ tract. The irreversible phase transition of Atx2-N317 caused by longer polyQ lengths (33Q and 41Q) is consistent with the protein aggregation observed under the pathological condition of SCA2.

### 2.2. Status and Distribution of PolyQ-Expanded Atx2-N81 in Cells

Atx2-N317 contains a polyQ tract prone to aggregation and the LSm-LSmAD domains involved in protein–RNA interactions [28]. Previous studies have shown that the LSm domain of Atx2 inhibits phase separation from the LSmAD domain, and the Atx2 mutants with the deleted LSm-LSmAD domains are more prone to aggregation in cells [21]. This implies that the phase separation and transition of Atx2-N317 are influenced by the polyQ sequence effectively. By using the FuzDrop method [40], we predicted the regions that have a propensity to phase separation and aggregation in the Atx2-N317 sequence (Figure S2). It showed that the N-terminal segment (residues 1–94) with the polyQ tract was included in the droplet-promoting region (DPR), with a droplet promotion score (P_DP_) generally above 0.9. Moreover, this sequence also had two hot spots (residues 13–20 and 80–92) for aggregation. This suggests that the polyQ region may strongly promote biomolecular condensation and aggregation in the Atx2 protein.

**Figure 3:**
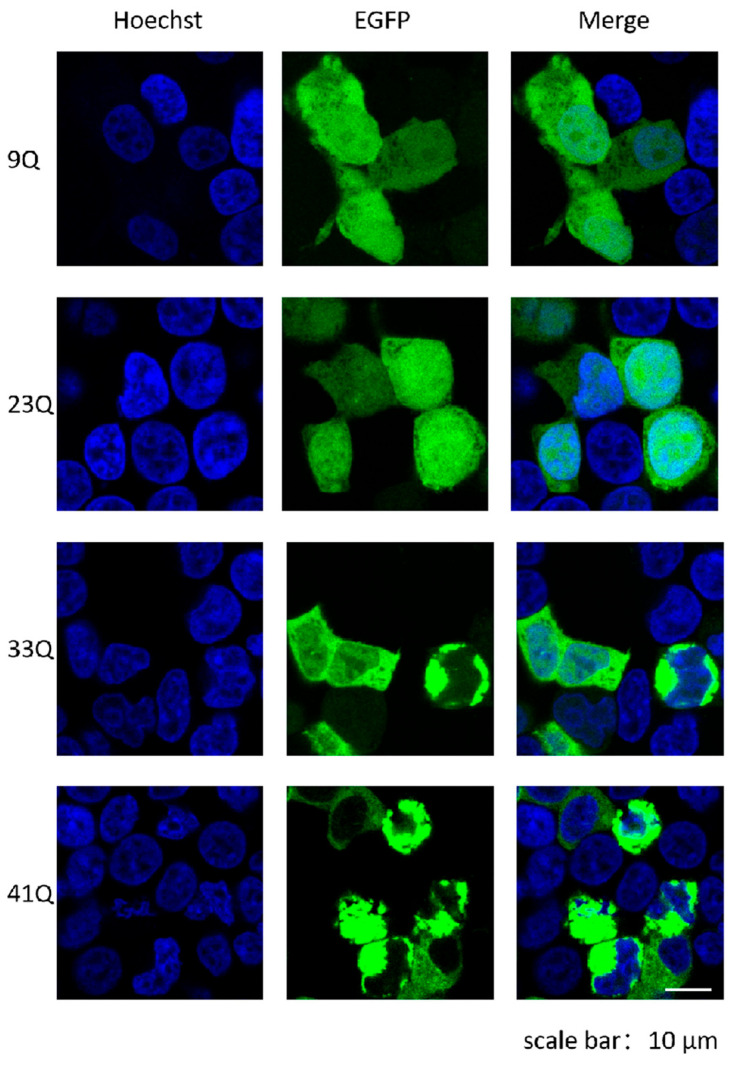
Immunofluorescence imaging showing the status and subcellular distribution of Atx2-N81 with different polyQ expansions. About 48 h after the transfection of the respective EGFP-Atx2-N81 plasmids in HEK 293T cells, the cells were fixed and photographed. Green, Atx2-N81 imaged with EGFP; blue, nuclei stained with Hoechst. Scale bar, 10 μm. 9Q, EGFP-Atx2_9Q_-N81; 23Q, EGFP-Atx2_23Q_-N81; 33Q, EGFP-Atx2_33Q_-N81; 41Q, EGFP-Atx2_41Q_-N81.

To investigate the effect of polyQ on the molecular status and mobility of Atx2, we constructed a series of EGFP-fused Atx2-N81 fragments with different polyQ lengths. Immunofluorescence imaging exhibited that Atx2_9Q_-N81 and Atx2_23Q_-N81 were diffusely distributed in cells, with a few molecules of Atx2_23Q_-N81 forming small spots within cells (Figure 3). While Atx2_33Q_-N81 formed some puncta in the cytoplasm, Atx2_41Q_-N81 was mainly distributed in the cytoplasm, forming larger puncta occupying most of the space in the cells. These results demonstrate that polyQ expansion promotes the biomolecular condensation and aggregation of Atx2-N81 as well as Atx2-N317.

### 2.3. Molecular Mobility of Atx2-N81 with Different PolyQ Expansions

**Figure 4:**
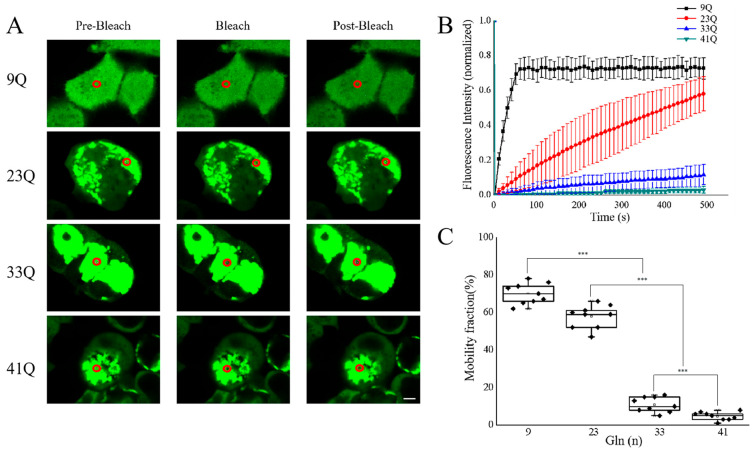
Molecular mobilities of Atx2-N81 with different polyQ expansions. (**A**) FRAP analysis for the molecular mobilities of Atx2-N81 with different polyQ lengths. HEK 293T cells were transfected with the respective EGFP-Atx2-N81 plasmids, and after a 48 h culture, the cells were subjected to FRAP experiments. The red circle represents the range of photobleaching. The changes in fluorescence intensity at the bleaching site were recorded at the end of photobleaching, and the time was set as the starting point. Scale bar, 2 μm. 9Q, EGFP-Atx2_9Q_-N81; 23Q, EGFP-Atx2_23Q_-N81; 33Q, EGFP-Atx2_33Q_-N81; 41Q, EGFP-Atx2_41Q_-N81. (**B**) Fluorescence recovery profile after photobleaching. The curve exhibits the fluorescence intensity recovering at the bleaching site over time, normalized with the fluorescence intensity of the spot before photobleaching. (**C**) Fluorescence recovery rate of photobleaching. Data are from three repeats and represented as the Mean ± SEM. Statistical analysis was carried out with ANOVA. ***, *p* < 0.001.

To further investigate the mobility of the protein puncta formed by Atx2-N81 with different polyQ expansions, we performed FRAP experiments on the different puncta in HEK 293T cells (Figure 4A). Atx2_9Q_-N81 had its fluorescence recovered immediately after photobleaching, indicative of its relatively high molecular mobility in cells. Atx2_23Q_-N81 retained some mobility, and its fluorescence recovery reached 60% 300 s after bleaching, whereas Atx2_33Q_-N81 and Atx2_41Q_-N81 almost lost their mobilities, with the respective recovery levels of only 10% and 5% (Figure 4B,C). So, the FRAP data suggest that the Atx2-N81 protein remains completely soluble and dynamic in cells when the polyQ tract is rather short. It may undergo phase separation in the cytoplasm, forming highly dynamic droplet particles capable of molecular exchange with the surrounding environment. However, when polyQ expands to higher repeats, the fluorescence in the ROIs of the puncta cannot be recovered, indicating that the Atx2-N81 protein forms solid-like aggregates or particles without any mobility. Moreover, the solidification extents of the particles increase as the number of glutamine residues increases. This is consistent with the pathogenic nature of Atx2, that is, the more polyQ expansion in the Atx2 protein, the earlier and more severe the disease.

### 2.4. PolyQ Expansion Promotes Biomolecular Condensation and Aggregation of Atx2-N81 In Vitro

**Figure 5:**
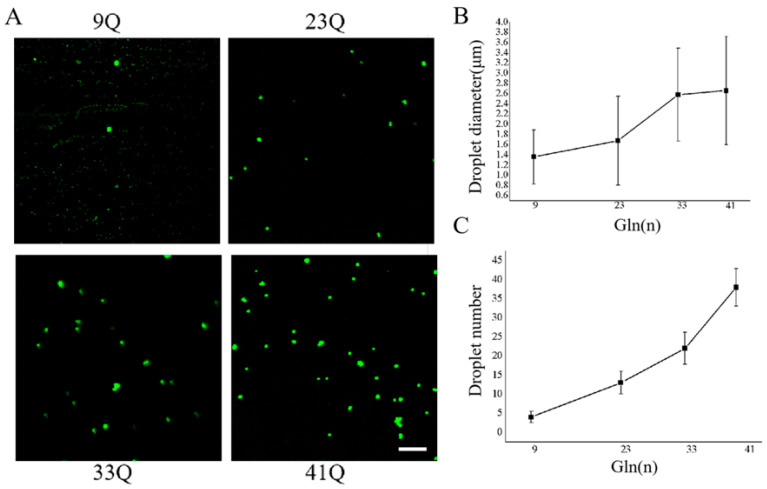
PolyQ expansion promotes the phase separation of Atx2-N81 in vitro. (**A**) Droplet formation of EGFP-Atx2-N81 with different polyQ lengths in vitro. The EGFP-Atx2-N81 proteins (5 μM) were incubated in a PBS solution containing 10% PEG8000 for 30 min; then, images of droplet formation in the chamber were taken under a confocal microscope. Scale bar, 20 μm. 9Q, EGFP-Atx2_9Q_-N81; 23Q, EGFP-Atx2_23Q_-N81; 33Q, EGFP-Atx2_33Q_-N81; 41Q, EGFP-Atx2_41Q_-N81. (**B**,**C**) Quantification of the droplet number (**B**) and diameter (**C**) in (**A**). Data are from three repeats and represented as the Mean ± SEM.

To further verify the impact of polyQ expansion on the protein condensation and aggregation in Atx2, we constructed four plasmids for the prokaryotic expression of the EGFP-tagged Atx2-N81 species and purified these proteins for in vitro studies (Figure S3). Under a phosphate buffer system (10 mM Na_2_HPO_4_, 2 mM KH_2_PO_4_, 2.7 mM KCl, 150 mM NaCl, 10% PEG 8000, and pH 7.4), all four Atx2-N81 species can undergo phase separation in solution, forming droplet-like condensate structures. We compared the abilities of these Atx2-N81 species in phase separation and formation of droplets at the protein concentration of 5 μM (Figure 5A). The results showed that the number of droplets formed by Atx2-N81 significantly increased with the polyQ expansion (Figure 5B). Statistical analysis of the droplet size revealed that the average droplet diameter from 33Q or 41Q was significantly larger than that from 9Q or 23Q (Figure 5C). The profile of droplet diameter versus the Q number suggests that there may be a transition turn point around 23Q–33Q residues from condensation to aggregation. This study confirms that the condensation capability of Atx2-N81 in vitro is proportional to the degree of glutamine repeats, which may be consistent with the pathological mechanism of phase transition from physiological conditions.

**Figure 6:**
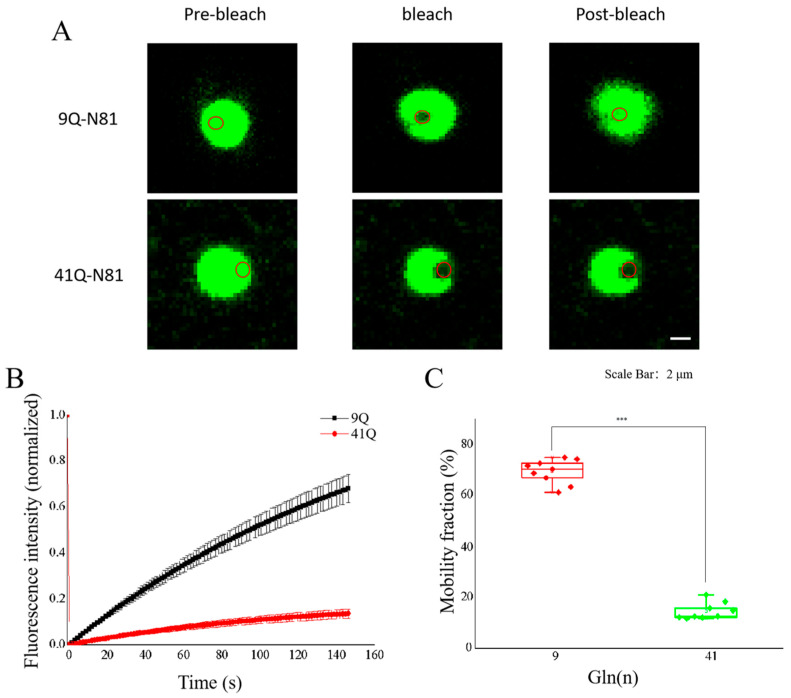
Molecular mobilities of the EGFP-Atx2-N81 droplets in vitro. (**A**) FRAP detection of the molecular mobilities of the droplets formed by 9Q or 41Q EGFP-Atx2-N81 in vitro. Each EGFP-Atx2-N81 protein (5 μM) was incubated in the PBS solution containing 10% PEG 8000 for 30 min; then, the FRAP experiment was carried out. The red circle represents the range of photobleaching. The changes in fluorescence intensity at the bleaching site were recorded at the end of photobleaching, and the time was set as the starting point. Scale bar, 2 μm. 9Q, EGFP-Atx2_9Q_-N81; 41Q, EGFP-Atx2_41Q_-N81. (**B**) Fluorescence recovery profile after photobleaching. The curve shows the fluorescence intensity recovering at the bleaching site over time, normalized with the fluorescence intensity of the spot before bleaching. (**C**) Fluorescence recovery rate of photobleaching. The data from three repeats were analyzed with ANOVA and are represented as the Mean ± SEM. ***, *p* < 0.001.

To compare the fluidity of the droplets formed by Atx2-N81 with different polyQ expansions, we performed a FRAP experiment on the droplets formed by Atx2-N81 with 9Q or 41Q (Figure 6A). The results showed that the fluorescence intensity of the Atx2_9Q_-N81 puncta could recover to 80% within 150 s after bleaching, whereas the fluorescence recovery rate of Atx2_41Q_-N81 was only 10% (Figure 6B,C). This indicates that Atx2-N81 forms droplet-like condensates in a short polyQ tract, while it transforms into solid-like aggregates with the expansion of this tract. The molecular mobility of Atx2-N81 with a short polyQ tract is significantly higher than that with a longer polyQ, further demonstrating that polyQ expansion controls the molecular dynamics of Atx2 and even other polyQ proteins.

## 3. Discussion

As a special RBP, Atx2 possesses RNA-binding properties that play a role in RNA metabolism and homeostasis, but the polyQ tract in its N-terminus also plays an important role in biomolecular condensation and aggregation, which are associated with cellular homeostasis and disease pathology. We studied two N-terminal fragments, Atx2-N317 and Atx2-N81, with different polyQ-tract lengths, and found that these two fragments have little difference in their biomolecular behaviors in cells. In the over-expression scenarios, the protein state, cellular distribution, and biomolecular mobility of Atx2-N81 are largely consistent with those of Atx2-N317. This suggests that polyQ is a sufficient factor for condensation of the N-terminal portion in Atx2, and polyQ expansion can enhance its phase separation ability. The polyQ tract with the repeat lengths exceeding a certain threshold is the primary cause of the phase transition and formation of insoluble amyloid aggregates. The LSm and LSmAD domains outside the polyQ tract may participate in the phase separation of Atx2 through RNA-mediated protein interactions, but this process is probably independent of the molecular mobility of the Atx2 protein itself.

Atx2-N81 contains only a polyQ tract but without LSm and LSmAD domains. However, similar to Atx2-N317, it can also undergo biomolecular condensation in cells, forming droplets with a certain extent of molecular mobility. With the polyQ expansion, the mobility of Atx2-N81 gradually decreases, leading to the irreversible transformation of the protein into solid aggregates. This means that the polyQ tract itself is sufficient for protein condensation and aggregation, and its ability to undergo phase transition increases with the expansion of polyQ. When the polyQ length exceeds a threshold (e.g., 33Q), Atx2-N81 may undergo liquid–solid phase transition to aggregates. This implies that, in full-length Atx2, the tendency for phase separation is inherent to the protein itself but may not depend on protein–protein/RNA interactions under some conditions. However, because the phase separation ability of the polyQ tract itself is positively correlated with the number of glutamine repeats, Atx2 readily promotes liquid–solid phase transition, resulting in insoluble protein aggregates or inclusions in cells [41].

We have previously verified that both Atx2 and its Atx2-N317 fragment exhibit similar solubility behaviors with polyQ expansions [26]. Compared to the full-length Atx2, Atx2-N317 lacks the C-terminal IDRs and the PAM2 domain. Previous studies have shown that the C-terminal part of Atx2 is involved in the phase separation process of Atx2 in cells, forming membrane-less organelles [42]. Both Atx2 and Atx2-N317 are soluble under the normal condition of less than 23Q in cells, while with the polyQ expansion, the insoluble aggregates formed by Atx2-N317, as well as by Atx2, gradually increase. At the same time, due to the presence of LSm and LSmAD domains that may interact with RNA, Atx2-N317, similar to full-length Atx2, mostly retains its biochemical properties, such as interactions with and the sequestration of DDX6 when polyQ is abnormally expanded [26]. Atx2 also contains the C-terminal IDRs with a PAM2 motif that may interact with PABPC1 and poly(A) of mRNA [23]. As a research model, Atx2-N317 provides an example recapitulating the biomolecular behaviors and dynamics in the absence of intracellular interactions with PABPC1 as well as poly(A) tails of mRNA. Although Atx2-N317 lost its ability to interact with PABPC1 or possibly with poly(A) of mRNA, it still possessed the ability for phase separation and formation of SGs, depending on the polyQ-tract length in its N-terminus. Moreover, Atx2-N81 lost its LSm and LSmAD domains, but it also had the potential to form biomolecular condensates and even aggregates. This suggests that the polyQ tract in Atx2 contains intrinsic properties that confer the protein a capacity for biomolecular condensation and aggregation.

In summary, this study demonstrates that the polyQ tract and its abnormal expansion control the biomolecular condensation and aggregation of the N-terminal fragments of Atx2, ruling out the interference from the LSm and LSmAD domains outside. This will challenge the previous understanding that the biomolecular condensation and aggregation of Atx2 are mediated by RNA binding and the C-terminal IDR sequences. When the polyQ tract expands to a certain extent, Atx2 may undergo a phase transition from a reversible condensation process to an irreversible insoluble aggregation.

## 4. Materials and Methods

### 4.1. Plasmids and Reagents

To clone the N-terminal truncations, the cDNAs encoding the sequences of Atx2-N317 and Atx2-N81 were amplified via PCR from the pcDNA3.1-FLAG-Atx2 plasmid [43] using the primers of Atx2-F and Atx2-N317-R or Atx2-N81-R (Table S1), and the EFGP cDNA was amplified from the pEGFP-C1 plasmid using the primers of EGFP-F and EGFP-Atx2-R. The DNA sequences encoding Atx2-N317 and Atx2-N81 and their variant species with different Gln numbers (9Q, 23Q, 33Q, and 41Q) were cloned into a pEGFP-C1 vector via Xho I/EcoR I sites. For prokaryotic expression, the DNA sequences encoding Atx2-N81 or its variants (9Q, 23Q, 33Q, and 41Q) and the EGFP sequence were cloned into a pET22b vector via Nde I/BamH I/Not I sites. All the constructs were verified by DNA sequencing (Table S2).

### 4.2. Cell Culture and Plasmid Transfection

The HEK 293T cell line (RRID: CVCL_1926) was from the Cell Bank of the Chinese Academy of Sciences (Shanghai, China). The cell culture was carried out in a DMEM medium (Corning, Glendale, CA, USA) supplemented with 10% FBS (BioInd, Kibbutz Beit-Haemek, Israel) and penicillin/streptomycin at 37 °C under a humidified atmosphere with 5% (*v*/*v*) CO_2_. Plasmid transfection was conducted with a PolyJet reagent (SignaGen, Rockville, MD, USA) according to the manufacturer’s instructions. First, the plasmid solution was mixed with the diluted PolyJet reagent and incubated at room temperature for 15 min. Then, the mixture was added to the medium and further cultured for 8 h. Lastly, the original medium was replaced with a fresh complete medium.

### 4.3. Immunofluorescence Imaging

About 48 h after transfection with the indicated plasmid, the cells grown on glass slips were washed with a PBS buffer (10 mM Na_2_HPO_4_, 1.8 mM KH_2_PO_4_, 140 mM NaCl, 2.7 mM KCl, and pH 7.4). Then, the cells were fixed with 4% paraformaldehyde at 4 °C for 15 min, permeabilized with 0.1% Triton X-100, and blocked with the blocking solution (10% BSA in the PBS buffer) for 1 h at room temperature. The fixed cells were then incubated with a specific primary antibody at 4 °C overnight, and after being washed with the PBS buffer 3 times, the cells were labeled with FITC- or TRITC-conjugated antibody (Jackson ImmunoResearch Laboratories, West Grove, PA, USA). Hoechst 33342 (ThermoFisher Scientific, Rockford, IL, USA) was applied to stain the nuclei. Finally, the cells were visualized on a confocal microscope (TCS SP8 WLL, Leica Microsystems, Wetzlar, Germany).

### 4.4. Purification of EGFP-Atx2-N81 Proteins

The plasmids for expressing the respective EGFP-Atx2-N81 species were transformed into BL21 (DE3) competent *Escherichia coli* cells. When the cells were grown at 37 °C to an A600 of 0.6∼0.8, IPTG was added to a final concentration of 0.2 mM and continuously cultured at 22 °C overnight. After protein expression, the cells were harvested by centrifugation and lysed by sonication with a lysis buffer (50 mM NaH_2_PO_4_, pH 8.0, 500 mM NaCl, and 1 mM PMSF). Then, the lysates were centrifuged at 12,000 rpm for 30 min at 4 °C, and the pellets were discarded. The supernatant was loaded onto an Ni^2+^-affinity column (Roche, Basel, Switzerland) and washed three times with the washing buffer (50 mM NaH_2_PO_4_, pH 8.0, 500 mM NaCl, and 25 mM imidazole), and then, the proteins were eluted using the elution buffer (50 mM NaH_2_PO_4_, pH 8.0, 500 mM NaCl, and 250 mM imidazole). Next, the protein eluates were concentrated using a 10-K centrifugal filter device (Millipore, Billerica, MA, USA) and further purified by FPLC Size-exclusion chromatography (SEC) with an analytical column Superdex^TM^-200 10/300 GL (GE Healthcare, Uppsala, Sweden). Lastly, the protein solution was collected and stored at 4 °C for use.

### 4.5. Phase Separation Experiment In Vitro

For the in vitro phase separation, the imaging chambers were prepared following the protocol described in [44]. Before droplet formation, the protein concentration was determined using a BCA Protein Assay kit. The protein was diluted to a suitable concentration (5 μM) in the PBS buffer containing 10% PEG 8000. Then, the protein solution was incubated at room temperature for 30 min, followed by centrifugation at 12,000 rpm for 3 min to remove any aggregates. Finally, the protein samples were put into the imaging chambers previously prepared and visualized by confocal microscopy (Leica TCS SP8 WLL, Leica Microsystems, Wetzlar, Germany).

### 4.6. Fluorescence Recovery After Photobleaching

To study molecular dynamics using the FRAP experiment, the HEK 293T cells were plated on a 29 mm dish with a 20 mm glass-bottom well (Cellvis, Mountain View, CA, USA). About 48 h after transfection with the N-terminal fragments of Atx2 or their variants, the growth media were replaced with fresh ones. Then, the cells were imaged on an inverted laser scanning confocal microscope (Olympus SpinSR, Olympus Corp., Hachioji-shi, Tokyo, Japan), with a random selection of a circular area of about 1.5 μm^2^ as the bleaching spot. Before bleaching, five frames were acquired as the baseline fluorescence of the bleaching spot using the 488 nm laser with 10% intensity. Next, the spots were bleached 50 times using a 405 nm laser with 20% intensity. After bleaching, the recovery of the fluorescence intensities was monitored every 5 s for 100 frames by confocal microscopy (Olympus SpinSR, Olympus Corp., Hachioji-shi, Tokyo, Japan).

For the FRAP experiment on the droplets formed in vitro by phase separation, a droplet with a circular area of about 0.5 μm^2^ was randomly selected as the bleaching spot, and before bleaching, five frames were acquired, with 2 s intervals as the baseline fluorescence of the bleaching spot, using a 488 nm laser with 15% intensity. Next, the droplet spots were bleached 20 times by the 488 nm laser with 90% intensity. After bleaching, the fluorescence recovery was monitored every 3 s for 50 frames by confocal microscopy (Leica TCS SP8 WLL, Leica Microsystems, Wetzlar, Germany).

## Data Availability

The original contributions presented in this study are included in the article/Supplementary Material. Further inquiries can be directed to the corresponding author.

## Supplementary Materials

The following supporting information can be downloaded at: https://www.mdpi.com/article/10.3390/ijms262311538/s1.

## Abbreviations

The following abbreviations are used in this manuscript:

ALS	Amyotrophic lateral sclerosis
Atx2	Ataxin-2
BCA	Bicinchoninic acid
BSA	Bovine serum albumin
EGFP	Enhanced green fluorescent protein
FBS	Fetal bovine serum
FITC	Fluorescein isothiocyanate
FPLC	Fast protein liquid chromatography
FRAP	Fluorescence recovery after photobleaching
LCD	Low-complexity domain
LSm	Like Sm
LSmAD	LSm-associated domain
PAM2	PABP-binding motif 2
P-body	Processing body
PBS	Phosphate-buffered saline
polyQ	polyglutamine;
RBP	RNA-binding protein
ROI	Region of interest
SCA2	Spinocerebellar ataxia type 2
SEC	Size-exclusion chromatography
SG	Stress granule
TRITC	Tetramethylrhodamine isothiocyanate
