# Supplementary material for: PolyQ Expansion Controls Biomolecular Condensation and Aggregation of the N-Terminal Fragments of Ataxin-2
Source: Int J Mol Sci. 2025 Nov 28;26(23):11538. doi: 10.3390/ijms262311538 (PMC12691732; doi:10.3390/ijms262311538)
Supplement: Supplementary file 1 [file ijms-26-11538-s001.zip › ijms-3938467-supplementary.pdf]

PolyQ Expansion Controls Biomolecular Condensation and Aggregation of the N-Terminal Fragments of Ataxin-2

Yin-Hu Liu [1,2], Heng-Tong Duan [1,2], Lei-Lei Jiang [1] and Hong-Yu Hu [1,*]

[1] Key Laboratory of RNA Innovation, Science and Engineering, Center for Excellence in Molecular Cell Science, Shanghai Institute of Biochemistry and Cell Biology, Chinese Academy of Sciences, Shanghai 200031, China; liuyinhu2021@sibcb.ac.cn (Y.-H.L.); duanhengtong2021@sibcb.ac.cn (H.-T.D.); jiangleilei@sibcb.ac.cn (L.-L.J.)
[2] University of Chinese Academy of Sciences, Beijing 100049, China
* Correspondence: hyhu@sibcb.ac.cn

Figure S1

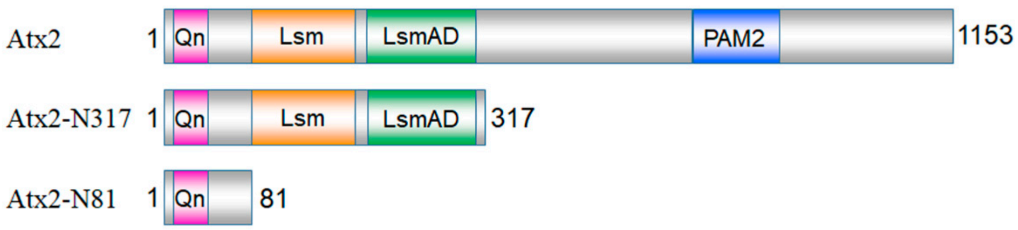

Figure S1. Domain architecture of Atx2 showing its N-terminal fragments applied in this study. Atx2-N317, residues 1-317 of Atx2; Atx2-N81, residues 1-81 of Atx2; Qn, polyQ tract; LSm, Like Sm domain; LSmAD, LSm-associated domain. PAM2, PABP-binding motif 2.

Figure S2

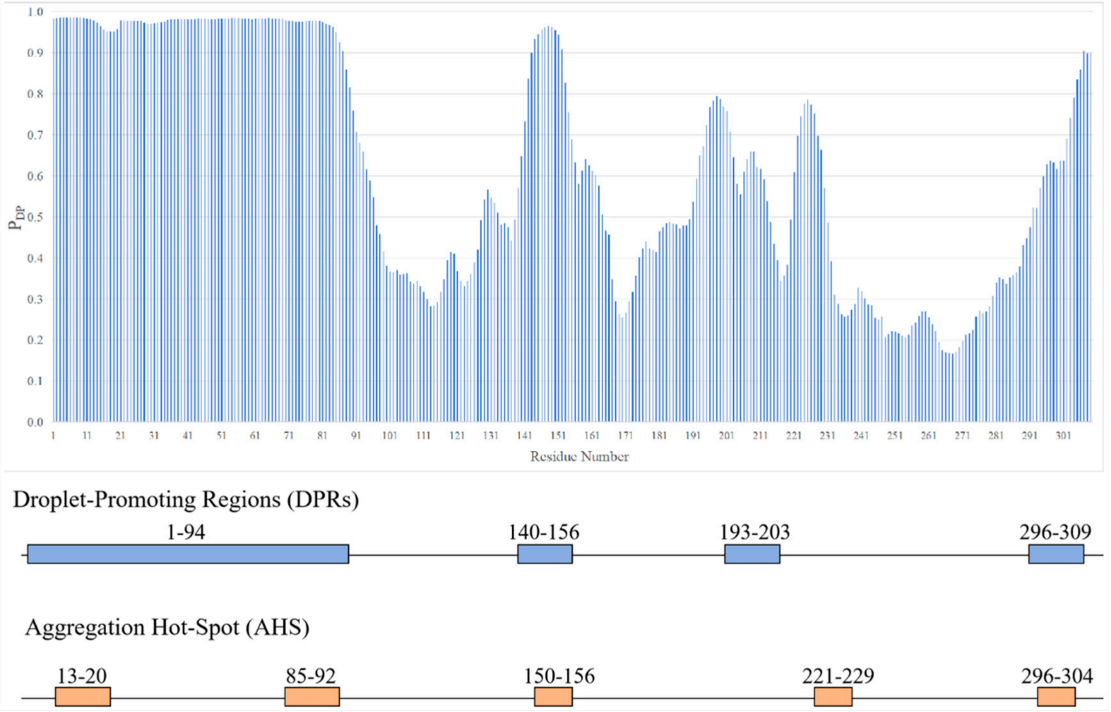

Figure S2. Prediction of the droplet-promoting regions in the Atx2-N317 sequence by the FuzDrop method. P_{DP}, droplet-promoting probability; DPR, droplet-promoting region; AHS, aggregation hotspot. FuzDrop server：https://fuzdrop.bio.unipd.it.

Figure S3

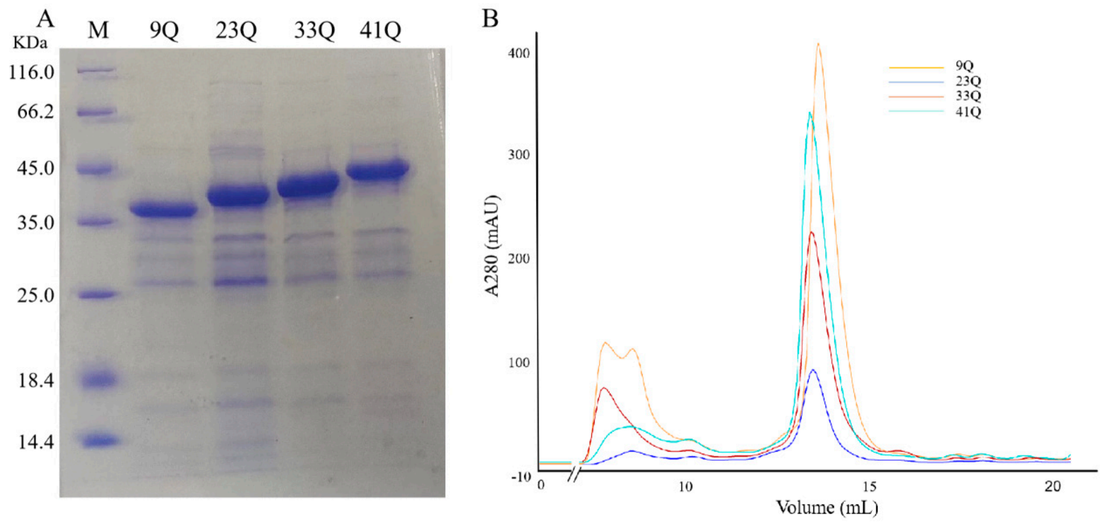

Figure S3. Purification of EGFP-Atx2-N81 with various polyQ expansions. (A) SDS-PAGE for examining the purification of EGFP-Atx2-N81 with various polyQ lengths. The proteins were loaded in gels for SDS-PAGE with Coomassie blue staining. M, standard protein marker. (B) Size exclusion chromatography (SEC) of EGFP-Atx2-N81 with various polyQ lengths. The peaks in the eluate volumes of 13-15 mL were collected and stored. 9Q, EGFP-Atx2$_{9Q}$-N81; 23Q, EGFP-Atx2$_{23Q}$-N81; 33Q, EGFP-Atx2$_{33Q}$-N81; 41Q, EGFP-Atx2$_{41Q}$-N81.

Table S1. Nucleotide sequences of the PCR primers applied in this study.

PCR primer	Sequence	Note
pET22b-EGFP-F	TAAGAAGGAGATATACATATGGTGAGCAAGGGCGAG	Nde I
EGFP-BamHI-R	GGATCCATCGATTCGAGATCTGAGTCCGGACTTGTA	BamH I
EGFP-BamHI-Atx2-F	GGATATCGGAATTAATTCGCCATGAGCCTGAAGCCCCAG	BamH I
pET22b-NotI-Atx2-N81-R	GGTGGTGGTGCTCGAGTGCttaTCCAGGTCTTCCTCCTCCG	Not I
GFPC1-XhoI-Atx2-F	GACTCAGATCTCGAGATGAGCCTGAAGCCC	Xho I
Atx2-N81-BamHI-R	AGATCCGGTGGATCCttaTCCAGGTCTTCCTCC	BamH I
Atx2-N317-BamHI-R	AGATCCGGTGGATCCTTAATTTCTCTGAAC	BamH I

Table S2. List of the constructs applied in this study.

Construct	Vector	Restriction enzyme site	Additional
pET22b-EGFP-Atx2$_{9Q}$-N81	pET22b	Nde I/BamH I/Not I	Residues: 1-81 of Atx2; 9 Gln
pET22b-EGFP-Atx2$_{23Q}$-N81	pET22b	Nde I/BamH I/Not I	Residues: 1-81 of Atx2; 23 Gln
pET22b-EGFP-Atx2$_{33Q}$-N81	pET22b	Nde I/BamH I/Not I	Residues: 1-81 of Atx2; 33 Gln
pET22b-EGFP-Atx2$_{41Q}$-N81	pET22b	Nde I/BamH I/Not I	Residues: 1-81 of Atx2; 41 Gln
pEGFP-C1-Atx2$_{9Q}$-N81	pEGFP-C1	Xho I/BamH I	Residues: 1-81 of Atx2; 9 Gln
pEGFP-C1-Atx2$_{23Q}$-N81	pEGFP-C1	Xho I/BamH I	Residues: 1-81 of Atx2; 23 Gln
pEGFP-C1-Atx2$_{33Q}$-N81	pEGFP-C1	Xho I/BamH I	Residues: 1-81 of Atx2; 33 Gln
pEGFP-C1-Atx2$_{41Q}$-N81	pEGFP-C1	Xho I/BamH I	Residues: 1-81 of Atx2; 41 Gln
pEGFP-C1-Atx2$_{9Q}$-N317	pEGFP-C1	Xho I/BamH I	Residues: 1-81 of Atx2; 9 Gln
pEGFP-C1-Atx2$_{23Q}$-N317	pEGFP-C1	Xho I/BamH I	Residues: 1-81 of Atx2; 23 Gln
pEGFP-C1-Atx2$_{33Q}$-N317	pEGFP-C1	Xho I/BamH I	Residues: 1-81 of Atx2; 33 Gln
pEGFP-C1-Atx2$_{41Q}$-N317	pEGFP-C1	Xho I/BamH I	Residues: 1-81 of Atx2; 41 Gln
